# World Antimalarial Resistance Network (WARN) III: Molecular markers for drug resistant malaria

**DOI:** 10.1186/1475-2875-6-121

**Published:** 2007-09-06

**Authors:** Christopher V Plowe, Cally Roper, John W Barnwell, Christian T Happi, Hema H Joshi, Wilfred Mbacham, Steven R Meshnick, Kefas Mugittu, Inbarani Naidoo, Ric N Price, Robert W Shafer, Carol H Sibley, Colin J Sutherland, Peter A Zimmerman, Philip J Rosenthal

**Affiliations:** 1 Center for Vaccine Development, University of Maryland School of Medicine, 685 West Baltimore Street, HSF1-480, Baltimore, Maryland 21201 USA; 2 London School of Hygiene and Tropical Medicine, Keppel Street, London, UK WC1E 7HT; 3 Division of Parasitic Diseases, National Center for Infectious Diseases, Centers for Disease Control and Prevention, 4770 Buford Highway, NE MS F-13, Atlanta, Georgia 30341 USA; 4 Department of Immunology and Infectious Diseases, Harvard School of Public Health, 665 Huntington Avenue, Boston, Massachusetts 02115 USA; 5 National Institute of Malaria Research, 22 Sham Nath Marg, Delhi 110054, India; 6 Biotechnology Centre, University of Yaounde I, Cameroon, Box 8094 Yaounde, Cameroon; 7 University of North Carolina Schools of Public Health and Medicine, Chapel Hill, North Carolina 27599 USA; 8 Ifakara Health Research and Development Centre, P. O. Box 53, Ifakara, Tanzania; 9 Malaria Research Programme, Medical Research Council, PO Box 70380, Overport, 4067, South Africa; 10 International Health Programme, Menzies School of Health Research, P. O. Box 41096, Casuarina, Darwin, NT 0811 Australia; 11 Division of Infectious Diseases, Room S-169, Stanford University Medical Center, 300 Pasteur Drive, Stanford, California 94305 USA; 12 Department of Genome Sciences, Box 355065, University of Washington, Seattle, Washington 98195 USA; 13 London School of Hygiene & Tropical Medicine, Department of Infectious and Tropical Diseases, Immunology Unit, Keppel Street, London WC1E 7HT, UK; 14 Case Western Reserve University, The Center for Global Health & Diseases, Wolstein Research Building, Room 4-125, 2103 Cornell Road, Cleveland, Ohio 44106 USA; 15 University of California San Francisco, Box 0811, San Francisco, California 94143 USA

## Abstract

Molecular markers for drug resistant malaria represent public health tools of great but mostly unrealized potential value. A key reason for the failure of molecular resistance markers to live up to their potential is that data on the their prevalence is scattered in disparate databases with no linkage to the clinical, in vitro and pharmacokinetic data that are needed to relate the genetic data to relevant phenotypes. The ongoing replacement of older monotherapies for malaria by new, more effective combination therapies presents an opportunity to create an open access database that brings together standardized data on molecular markers of drug resistant malaria from around the world. This paper presents a rationale for creating a global database of molecular markers for drug resistant malaria and for linking it to similar databases containing results from clinical trials of drug efficacy, in vitro studies of drug susceptibility, and pharmacokinetic studies of antimalarial drugs, in a World Antimalarial Resistance Network (WARN). This database will be a global resource, guiding the selection of first line drugs for treating uncomplicated malaria, for preventing malaria in travelers and for intermittent preventive treatment of malaria in pregnant women, infants and other vulnerable groups. Perhaps most important, a global database for molecular markers of drug resistant malaria will accelerate the identification and validation of markers for resistance to artemisinin-based combination therapies and, thereby, potentially prolong the useful therapeutic lives of these important new drugs.

## Background

This article is the result of a workshop held in October, 2006, at the Wellcome Trust Sanger Genome Centre in Hinxton, England. The background section reviews lessons learned over the last 15 years as molecular markers for drug resistant malaria were identified, validated, and to a regrettably limited degree, applied as tools to guide the use of drugs whose efficacy is now severely compromised by resistance. An argument is made that this history must not be repeated in the era of artemisinin-based combination therapies (ACTs) by using molecular markers only to chart passively the rise of resistance to this new generation of highly efficacious drugs. A rationale is provided for creating a global database for molecular markers of drug resistant malaria that will be linked to databases for malaria drug efficacy trials, in vitro drug resistance, and pharmacokinetics, and for using this network of databases (World Antimalarial Resistance Network, or WARN) proactively as a tool to monitor and deter resistance and to guide malaria treatment and prevention policies. Potential sources of data that would go into the database are described, followed by discussion of who will benefit from the creation of a tool for monitoring antimalarial drug resistance and facilitating rapid public health responses to changes in resistance profiles.

Molecular markers for drug resistant malaria are based on genetic changes that confer parasite resistance to drugs used to treat and prevent malaria. Polymorphisms in the *Plasmodium falciparum *chloroquine resistance transporter (PfCRT) confer resistance to chloroquine [1,2], and mutations in the P-glycoprotein homologue (Pgh1) encoded by *pfmdr1 *modulate this resistance [3]. Polymorphisms in *pfmdr1 *and amplifications of this gene also affect susceptibility to structurally unrelated antimalarial drugs, including mefloquine, artesunate, lumefantrine and quinine [4-6]. Polymorphisms in *P. falciparum *dihydrofolate reductase (DHFR) cause resistance to the antifolate drugs including pyrimethamine and other DHFR inhibitors, and polymorphisms in dihydropteroate synthase (DHPS) cause resistance to sulphadoxine and other sulphas and sulphones [7,8].

These molecular markers have been validated as tools for surveillance of resistance [9,10], and their potential value to policymakers has been demonstrated by their use to help control a malaria epidemic [11], to guide national malaria treatment policies [12] and to monitor changes in parasite drug susceptibility following changes in malaria drug treatment policy [13]. However, by the time the molecular markers for resistance to chloroquine and the antifolate combination sulphadoxine-pyrimethamine (SP) were established as tools for predicting clinical treatment outcomes, resistance had already severely compromised the efficacy of these drugs in most of the world. The emergence and spread of resistance to chloroquine and SP has led to recommendations that they be replaced with ACTs, which offer much-improved efficacy [14]. However, the development of resistance to artemisinins or their partner drugs may severely limit the utility of ACTs, and reliable markers for monitoring resistance to ACTs are needed.

### Development and validation of molecular markers

Candidate molecular markers for resistance to chloroquine, SP and mefloquine were identified by laborious molecular genetic approaches, including identifying parasite homologues of genes that mediate resistance in other organisms [8,15,16], and analysing the progeny of genetic crosses between sensitive and resistant parasites [1,7,17]. Differences in DNA sequence or gene expression between sensitive and resistant parasites were described, and point mutations, [1,7,18] and differences in gene expression or copy number [19,20] were evaluated for associations with in vitro resistance phenotypes. Causal relationships between molecular markers and in vitro resistance were then confirmed in genetic transformation studies in which DNA sequence substitutions conferred changes in the resistance phenotypes of cultured parasite clones [2,21];Triglia, 1998 596/id;Reed, 2000 1279/id} or in model systems such as yeast [22,23].

**Figure 1:**
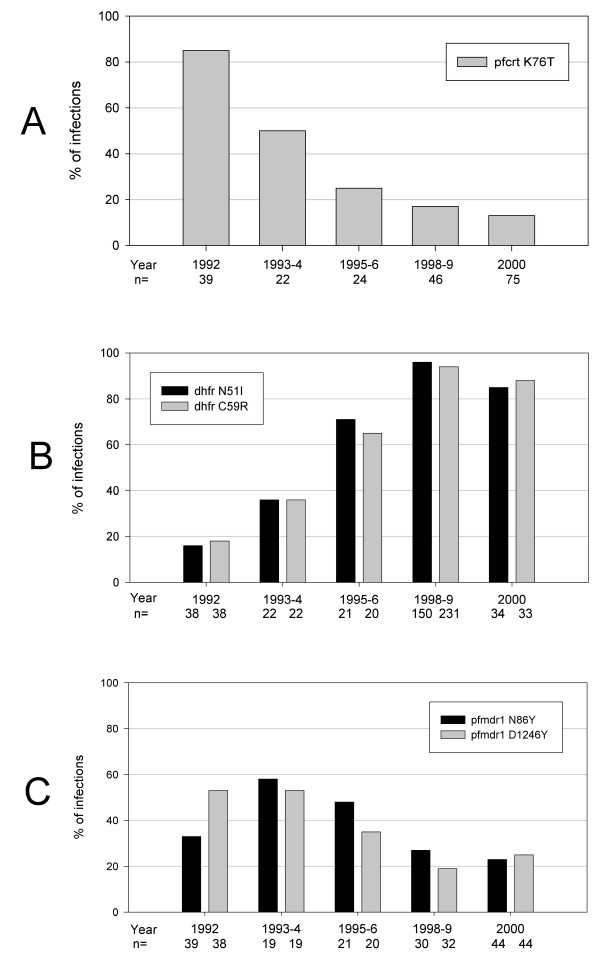
**Prevalence of sulphadoxine-pyrimethamine (SP) treatment failure and molecular markers of resistance to SP at five sites in Tanzania**. Ratios of prevalence of the DHFR triple mutant to SP treatment failure ranged from 2.0 to 2.1 at four of the five sites where SP resistance was low or moderate, suggesting that this molecular marker could serve as a reliable surrogate for SP efficacy at these sites. Adapted from [12] with permission.

To assess their clinical importance, molecular markers were evaluated in the field in ecological studies [24,25] and clinical trials [9,10,26]. Establishing the predictive value of molecular markers for outcomes of antimalarial drug treatment has been challenging, mainly because factors other than intrinsic parasite resistance affect these outcomes. Even for markers that correlate almost perfectly with in vitro resistance, other factors including acquired immunity [9,27], initial parasite biomass, compliance, dosing [28] and pharmacokinetics [20,29] affect the clearance of drug-resistant parasites. As the prevalence of resistance markers approaches fixation in a population, these non-resistance factors become more important in determining treatment outcomes [30]. The most important contributor to clearance of resistant parasites in high transmission areas is acquired immunity, which can be partially accounted for by controlling for age in a simple model for relating the prevalence of molecular markers to treatment failure rates [10,31]. When validated in a variety of settings with different levels of malaria transmission and acquired immunity and where resistance markers are not yet fixed in the population, this "genotype-failure index" model allows good prediction of treatment efficacy based on the prevalence of molecular markers for resistance (Figure 1). Unfortunately, without available pooled data from diverse sites, such as a global database for molecular markers would provide, this model has been validated only in a few local settings, including Mali [10], Uganda [32] and Tanzania [12] (Figure 1). Multivariate models that account for pharmacokinetic and other factors that contribute to treatment outcomes would improve the predictive ability of molecular markers for resistance, providing strong justification for the set of linked databases proposed in this series of papers. Such a comprehensive global database would provide the statistical power needed to assess the roles played by multiple genetic and non-genetic determinants of treatment outcomes.

Reliable methods for using molecular markers to monitor chloroquine and SP resistance were established only after resistance to these drugs was so widespread that the information provided by molecular surveys was, with some notable exceptions [11,12], mainly of historical interest and of little immediate relevance to antimalarial treatment policies. As ACTs become the first line in most countries, it will be important to characterize markers for resistance as it develops, before clinical efficacy is lost. Most efforts to date to identify the genetic determinants of resistance to the drugs that comprise ACTs have taken a candidate gene approach, based on homologues of resistance genes from other organisms or suspected targets for drug action. In an example of this approach, in vitro and clinical evidence suggests that increased *pfmdr1 *copy number is associated with decreased sensitivity to artemisinin and other antimalarial drugs [5,20]. Genes encoding potential targets of artemisinin action are also being pursued as molecular markers. Based on studies showing that artemisinins inhibit a Ca++ ATPase, sequence changes in the gene encoding PfATPAse6 were assayed and found to be associated with in vitro resistance in isolates from South America, but not Africa or Asia [33]. These candidate gene approaches rely on an optimistic hope that individual genes will be found to be responsible for resistance to ACTs. In the more likely scenario in which resistance to these drugs is mediated by multiple genes, whole-genome strategies that search for signatures of selection around resistance loci may accelerate the identification of molecular markers for resistance [34-36].

### Rationale for a global database of resistance markers

Clinical trials are and will remain the gold standard for measuring drug efficacy, but when assays for molecular resistance markers are sufficiently predictive of clinical treatment outcomes, their simplicity, robustness and scalability make them a potentially powerful adjunct to clinical trials. Molecular assays for common polymorphisms are straightforward and relatively low cost when scaled up. The assays generally evaluate DNA, so they can be performed from blood spots on filter paper, which are easy to collect and store [37]. Thus, samples can easily be collected by health workers when patients present for diagnosis and treatment of malaria, or in targeted cross-sectional surveys in areas with high prevalence of asymptomatic malaria infection. These molecular analyses are routinely performed in many laboratories in endemic countries and can be shipped internationally for quality control without a need for special containers or precautions.

### How resistance markers have been used

The potential public health value of molecular resistance markers lies in their utility as tools for surveillance of trends in parasite drug sensitivity. Routine use of molecular data in decisions regarding appropriate antimalarial therapy is not yet a reality, in part because of the lack of mechanisms for timely sharing of molecular data in formats that are useful for non-specialists. However, the direct practical value of molecular surveys was demonstrated in 1999 in Mali, where chloroquine was then the first line antimalarial treatment [11]. A sharp increase in the incidence of malaria in a non-immune population occurred in a district in northern Mali where clinical assessments of drug efficacy could not be made because of limited infrastructure and civil unrest. An epidemic outbreak investigation team collected slides for microscopic diagnosis and filter paper blood samples for molecular analyses. In the capital city, Bamako, microscopy and molecular assays for markers predictive of resistance to chloroquine and SP suggested an unexpectedly high prevalence of resistance to chloroquine, but not to SP. Based on this information, special efforts were made to obtain SP and the population was effectively treated. Had evaluation of the gene-specific molecular markers not been performed, chloroquine would have been used to try to contain the epidemic and considerable malaria morbidity and mortality would have been anticipated.

In another example of molecular markers informing treatment policy, when a replacement for chloroquine was urgently needed in Tanzania, the prevalence of SP resistance markers predicted that, while SP would initially be more effective than chloroquine, its efficacy would soon be compromised. Based on this information, the Ministry of Health adopted SP on an interim basis as the best available first line drug, while actively seeking a more effective regimen [12]. Molecular markers for resistance are also guiding malaria treatment policy in Southeast Asia, where initial reports of high rates of artesunate-mefloquine treatment failure on the Thai-Cambodian border [38,39] were met with skepticism about whether they represented resistance. However, when these treatment failures were shown to be highly correlated with *pfmdr1 *copy number [40], suggesting bona fide resistance to mefloquine, health officials began conducting surveys for the prevalence of this molecular marker and using this information to guide the choice of sites for in vivo assessments of efficacy.

**Figure 2:**
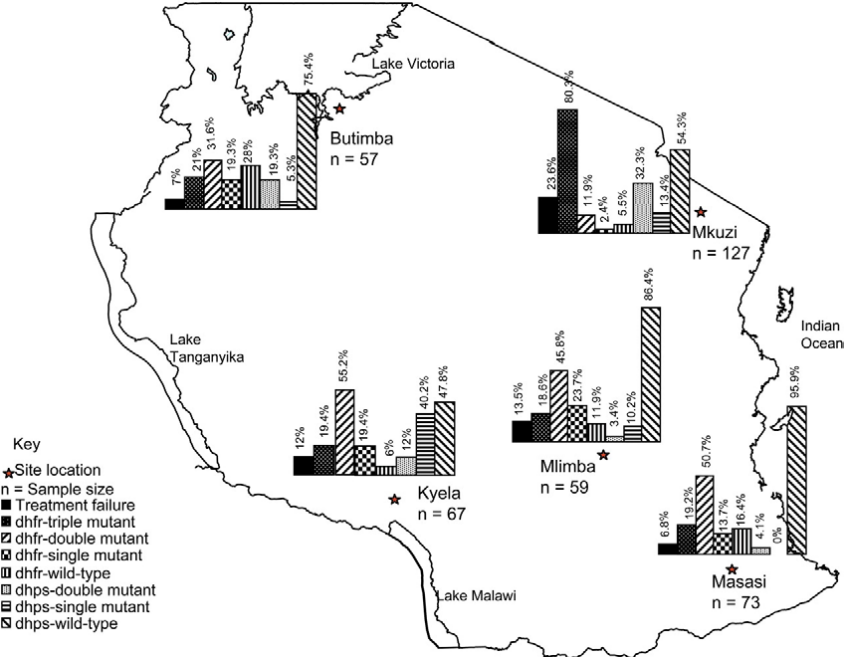
**Prevalence of drug-resistance mutations in *Plasmodium falciparum *malaria infections in Malawi before and after chloroquine was replaced with sulphadoxine-pyrimethamine in 1993**. Chloroquine resistance-conferring T76 mutation in PfCRT (A); Pyrimethamine resistance-conferring C59R mutations in DHFR (B); Adapted from [13] with permission.

Studies in Malawi illustrate the power of molecular resistance markers to track changes in drug susceptibility following changes in drug use policies. In 1993, Malawi became the first African nation to replace chloroquine with SP as the first line antimalarial drug countrywide [41]. Molecular surveillance of the PfCRT, DHFR and DHPS markers demonstrated two clear trends. First, the prevalence of the PfCRT T76 allele, which is associated with chloroquine resistance, declined rapidly after the withdrawal of chloroquine, from 85% in 1992 to undetectable levels by 2001. Second, the prevalence of parasites that carried DHFR mutations associated with resistance to SP progressively increased [1] (Figure 2). A clinical trial recently confirmed that these changes in the prevalence of resistance-mediating mutations have been accompanied by a dramatic increase in chloroquine efficacy in Malawi for the treatment of malaria, from about 50% to 99% in just 12 years, and an equally dramatic decrease in SP efficacy, from nearly 100% to 21% during the same time period [42].

### Why markers for chloroquine and SP resistance are still useful

Today, although the efficacies of chloroquine and SP have declined to unacceptable levels in much of Africa, attention to parasite mutations that mediate resistance to these drugs remains relevant for several reasons. First, in some areas the older drugs remain efficacious, including SP in parts of West Africa, and the combination of amodiaquine and SP in many areas [43,44]. Because they are inexpensive (about $0.20 U.S. per treatment), safe, and given in simple one- or three-day regimens, these drugs are likely to continue to be used even in the face of expert consensus that ACTs should be the first line therapy. However, little support will be available for continued clinical trials of chloroquine or SP efficacy under programmes funded by donors who recommend exclusive use of ACTs. Molecular surveillance that shows increasing prevalence of resistance markers known to be associated with treatment failure may be the only evidence available to convince policymakers that it is time to stop using these older drugs as their efficacy declines.

Second, SP is now recommended and increasingly used as intermittent preventive therapy (IPT) to prevent malaria in pregnant women [45] and infants [46], even in areas where SP efficacy for treatment of acute malaria is compromised. The value of mutations in DHFR and DHPS for predicting efficacy of IPT with SP needs to be established in settings with different levels of malaria transmission and acquired immunity, because of the impact of these factors on the relationship between prevalence of molecular markers and clinical efficacy. A global database of molecular markers for resistance will aid and accelerate the process of validating simple models for using these markers to predict SP efficacy for IPT, and then provide a means for monitoring this efficacy to guide decisions to move to newer but more expensive drugs as these are brought on line.

Third, an antifolate drug that is closely related to SP, trimethoprim-sulphamethoxazole, is a standard treatment for children with fever attributable to either respiratory infections or malaria [47], and is recommended as prophylaxis to prevent opportunistic infections in HIV-infected individuals in developing countries [48]. Trimethoprim-sulphamethoxazole prophylaxis effectively prevents malaria [49], and has the same genetic basis of in vitro resistance as SP [50,51]. However, it has been difficult to discern the impact that the DHFR and DHPS mutations that mediate resistance to SP may have on the protective efficacy of trimethoprim-sulphamethoxazole [49,52]. Surveillance for these mutations and pooled analysis of data from genotype and efficacy studies will clarify these associations. With a better understanding of relevant associations, coordinated surveillance for DHFR and DHPS mutations will be very helpful in determining optimal management strategies for people living with HIV in malaria endemic regions [53].

The fourth and final reason for continued surveillance of markers for resistance to chloroquine and SP is that, as was recently demonstrated in Malawi, the withdrawal of antimalarial drugs from use in a region may be followed by a return of clinical efficacy, heralded by falling prevalence of molecular markers for resistance [13,42]. Thus, surveillance of markers of resistance may help to guide the reintroduction of drugs whose efficacy has returned, in new combinations designed to deter the reemergence of resistance, thus creating the possibility of rotating drugs to maintain their efficacy [54].

### Molecular markers in the ACT era

As malaria treatment moves into the era of ACTs, the most important rationale for a database of molecular markers of drug resistance will be surveillance for resistance to the drugs included in these combinations. ACTs were designed to deter resistance by attacking parasites simultaneously with two or more drugs with different mechanisms of action, reducing the probability of resistance emerging [14]. These drug combinations were developed in settings of low malaria transmission, where the pharmacokinetic mismatch between the short-acting artemisinins and longer-acting partner drugs was not problematic, due to the low risk of encountering new infections during the elimination phase of the longer-acting partner drug. As these drugs are deployed in Africa, where the risk of new infection soon after treatment is high, the ability of the artemisinins to protect partner drugs from resistance is diminished. Thus, as ACTs are rolled out as first-line therapies for malaria, surveillance of molecular markers should be used to monitor the development of resistance to ACT partner drugs.

Each ACT partner drug is likely to select for resistance, potentially leading to loss of treatment efficacy as well as failure to protect the artemisinins against the development of resistance. At present the most important partner drugs used with artemisinins in ACTs are amodiaquine, lumefantrine, and piperaquine. The molecular mediators of resistance are not as well defined for these drugs as they are for chloroquine and SP, but recent data show hints of mechanisms of resistance. For amodiaquine, polymorphisms in both PfCRT and Pgh1 appear to predict resistance and to be selected for by treatment with amodiaquine [55] or artesunate-amodiaquine [56]. Artemether-lumefantrine treatment selects for polymorphisms in *pfmdr1 *associated with diminished sensitivity to the related drug halofantrine [57,58]. Markers for piperaquine resistance have not been identified, but this aminoquinoline may well act similarly to chloroquine and amodiaquine in its selection of resistance-mediating mutations.

To avoid unacceptably long delays in identifying, validating and deploying molecular markers of ACT resistance, the malaria research and control community must be prepared to investigate aggressively early reports of resistance, confirm resistance with careful in vitro assays, and bring genetic and genomic tools to bear to elucidate mechanisms and identify candidate molecular markers. The sequencing of the *P. falciparum *genome has led to genome-wide approaches that may help to identify genetic markers of drug resistance far more quickly than was previously possible [35,59,60]. There is a critical need to detect and confirm resistance to the component drugs in ACTs, and to the artemisinins in particular, as soon as it emerges, and then to develop and validate tools to monitor this resistance. These tools can then be applied in real time to help establish rational treatment policies and to design and deploy drug combinations that will deter resistance. The creation of a global database of molecular markers for drug resistance is a critical step in this process.

To the extent that known markers for resistance to ACT partner drugs are available now, a global database for collating and analysing trends in their prevalence will inform choices of which ACTs to introduce as countries change their policies. Moreover, a database of the type proposed will accelerate the process of validating predictive genotype-failure index models for ACT partner drugs in different settings with varying levels of malaria transmission and acquired immunity. Even more important, without a global database of molecular markers for drug resistant malaria, the evaluation of candidate molecular markers for resistance to the artemisinins and their partner drugs and the discovery of new such markers through whole-genome analyses is certain to lag far behind the development of resistance to these drugs.

### Sources of data

#### Drug efficacy trials

The data included in a global database of molecular markers for drug resistant malaria will consist primarily of the results of molecular assays for resistance markers. These will come from several sources. First, in most clinical trials of antimalarial drug efficacy, filter paper blood samples are routinely collected to preserve parasite DNA at the time of treatment and whenever post-treatment infections appear. These samples are usually subjected to analysis for genetic markers to determine whether post-treatment infections are due to recrudescence or to new infections, and in many cases the samples are also analysed for the presence of resistance markers. In studies aimed at validating candidate markers for resistance, these samples can be used to ask two types of questions: (1) Are resistance markers more common in post-treatment infections than in the initial infections, i.e. does drug treatment select for specific markers? (2) Is the presence of molecular markers for resistance at the time of presentation with malaria associated with the clinical and parasitological outcome of drug treatment, i.e. can markers predict efficacy? The first question is an important step in assessing putative markers for biological evidence of their association with resistance in vivo. The second question is a key step in validating the utility of molecular markers for predicting clinically relevant outcomes.

#### Molecular surveillance

In addition to surveys and efficacy trials in endemic areas, travelers returning from these areas to developed countries can provide surveillance data on drug resistant malaria [61,62]. Data from this source would provide useful information for travel medicine specialists and also serve as a sentinel surveillance system to direct targeted investigation of resistance [63]. Genotyping of isolates also subjected to in vitro drug susceptibility testing will provide another source of valuable data. These in vitro genotype-phenotype studies will be a crucial component of investigations to confirm and characterize reports of clinical resistance to ACTs, because they assess intrinsic drug resistance in the absence of confounding clinical factors such as host immunity. Lastly, data will come from the use of molecular markers as a broad surveillance tool. Once the association between resistance markers and clinical outcomes is well established, evaluation of samples collected in simple community surveys and at points of malaria diagnosis can provide a "snapshot" of local resistance patterns.

#### The need for standardization and linkage

Although standardized methods for recording and interpreting molecular resistance results have been proposed [26], a wide variety of approaches is presently used to analyse, interpret and report these data. Making the ability to access large datasets contingent on contributing data that adhere to standard formats will provide a strong incentive for investigators to do so, and should vastly increase the value of the large volumes of data that are presently available, but unlinked. For data from any of these sources, it will be essential to build systems that link the molecular data to corresponding demographic, geographic, epidemiological, clinical, in vitro and pharmacokinetic data. To insure that data are comparable and interpretable, it will be important to standardize genotyping technologies as well as data analysis and reporting. To this end reference laboratories should be supported to maintain and update standardized protocols on the network, and to provide blinded quality assurance testing of samples sent by contributing groups. Standardized reagents and controls can be provided by centralized references centers [64] or regional reference laboratories.

#### Resistant vivax malaria

Chloroquine resistant *Plasmodium vivax *arose in Asia in the 1980s [65] and has since spread throughout significant parts of Asia and South America. Resistance to other drugs, including antifolates and primaquine, also occurs with *P. vivax*. Some progress has been made toward identifying molecular markers for drug resistant vivax malaria [66,67]. As this work progresses, a database for molecular markers of resistance could easily be adapted to incorporate data for *P. vivax*, and inclusion of these data would aid in the process of validating *P. vivax *resistance markers.

#### Existing datasets

A starting point for a new global database will be to link a number of valuable existing databases. The utility of this approach will be limited to those datasets that include individual treatment outcome data that are amenable to conversion to standardized formats. The East African Network for Monitoring Antimalarial Treatment (EANMAT), formed in 1997, was the first network for monitoring drug resistance in Africa, with the objective of providing a dynamic assessment of current antimalarial treatment using standardized protocols, and generating data to guide policy changes. Similar regional surveillance networks have been created linking neighboring countries in other regions of Africa and in Southeast Asia and South America. The Multilateral Initiative on Malaria initiated an Antimalarial Drug Resistance Network linking fives study sites in Africa with the plan to systematically assess specific gene mutations, in vitro drug susceptibility, drug pharmacokinetics, and clinical treatment responses. The Mapping Malaria Risk in Africa project [68] has also developed a drug resistance database, sourcing published and unpublished in vivo and molecular resistance data from as many sites in Africa as possible. A global database would have the potential to link many of these existing databases, and greatly increasing both their usefulness and their use.

### Who will benefit?

A variety of consumers from different sectors will use and benefit from this database. Policymakers in malaria-endemic countries represent a primary set of clients. As local authorities attempt to promote evidence-based management of malaria, high quality up-to-date data on drug efficacy are needed. Of greatest value will be results of controlled clinical trials of antimalarial drug efficacy. However, limited resources will not support the number of trials needed to provide comprehensive characterization of efficacy, forcing authorities to make country-wide policies based on data from a limited number of sites. Well-designed studies of molecular markers of drug resistance can help to fill this gap. A database rapidly cataloguing molecular data and relaying it to policymakers would greatly facilitate the use of these data to help guide malaria treatment policies. It will be important for the data to be presented in ways that are easy for those with a range of expertise to understand and use. The database will facilitate this process by providing a means for standardizing data analysis, interpretation and reporting. The regional surveillance groups described in the preceding section will be a second set of consumers who benefit from the database. A new database linking and strengthening these networks will facilitate efficient use of available molecular data and stimulate trans-region discussions to encourage rational treatment policies. Those advising travelers from malaria-free countries to endemic areas will benefit from a public access database that provides early signals of resistance. Finally, the malaria research community will also profit from a global database. Research to understand the roles of known polymorphisms in resistance, and most importantly to identify new mediators of resistance, will be greatly helped by the availability of data describing prevalences of markers of resistance and associations with treatment outcomes. The database would include portals for data entry and analysis, with gates controlled by contributors who could decide when to submit data to the central database and when to make it accessible to all users. Working out the information technology and intellectual property issues for such a database will be challenging, but not without precedent, and will benefit from advances in systems for data management and the non-commercial nature of almost all malaria research programs.

## Summary

Molecular markers for resistance to older malaria drugs were validated only after the efficacy of these drugs was severely compromised by resistance. The potential of these markers as tools for malaria control has not been fully realized, in large part because data on their prevalence is scattered in many published and unpublished datasets in non-standardized formats. The development and validation of molecular markers for new combination antimalarial therapies is a high priority. In this paper a case has been made for creating a global public access database for molecular markers of drug resistance, linked to similar databases containing data on clinical efficacy, in vitro susceptibility and pharmacokinetics of antimalarial drugs, together comprising a World Antimalarial Resistance Network. This database would accelerate the development of markers for resistance to artemisinin-based and other combination therapies and help to inform rational malaria treatment and prevention policies designed to prevent and contain drug resistant malaria.

## Abbreviations

ACT, artemisinin-based combination therapy

DHFR, dihydrofolate reductase

DHPS, dihydropteroate synthase

PfCRT, *Plasmodium falciparum *chloroquine resistance transporter

Pgh1, P-glycoprotein-1

SP, sulphadoxine-pyrimethamine

WARN, World Antimalarial Resistance Network

## Authors' contributions

All authors contributed to the discussions that gave rise to the manuscript. CVP and PJR wrote the first draft and edited the final manuscript. CR edited the manuscript which includes contributions from JWB, CTH, HHJ, WM, SRM, KM, IN, RNP, RWS, CHS, CJS and PAZ.
